# Conservation and divergence of microRNAs in *Populus*

**DOI:** 10.1186/1471-2164-8-481

**Published:** 2007-12-31

**Authors:** Abdelali Barakat, Phillip K Wall, Scott DiLoreto, Claude W dePamphilis, John E Carlson

**Affiliations:** 1 Department of Biology, Institute of Molecular Evolutionary Genetics, and The Huck Institutes of the Life Sciences, 403 Life Sciences Building, The Pennsylvania State University, University Park, PA 16802, USA; 2 The School of Forest Resources, Department of Horticulture, and Huck Institutes of the Life Sciences, Pennsylvania State University, 323 Forest Resources Building, University Park, PA 16802, USA

## Abstract

**Background:**

MicroRNAs (miRNAs) are small RNAs (sRNA) ~21 nucleotides in length that negatively control gene expression by cleaving or inhibiting the translation of target gene transcripts. miRNAs have been extensively analyzed in *Arabidopsis *and rice and partially investigated in other non-model plant species. To date, 109 and 62 miRNA families have been identified in *Arabidopsis *and rice respectively. However, only 33 miRNAs have been identified from the genome of the model tree species (*Populus trichocarpa*), of which 11 are *Populus *specific. The low number of miRNA families previously identified in *Populus*, compared with the number of families identified in *Arabidopsis *and rice, suggests that many miRNAs still remain to be discovered in *Populus*. In this study, we analyzed expressed small RNAs from leaves and vegetative buds of *Populus *using high throughput pyrosequencing.

**Results:**

Analysis of almost eighty thousand small RNA reads allowed us to identify 123 new sequences belonging to previously identified miRNA families as well as 48 new miRNA families that could be *Populus*-specific. Comparison of the organization of miRNA families in *Populus*, *Arabidopsis *and rice showed that miRNA family sizes were generally expanded in *Populus*. The putative targets of non-conserved miRNA include both previously identified targets as well as several new putative target genes involved in development, resistance to stress, and other cellular processes. Moreover, almost half of the genes predicted to be targeted by non-conserved miRNAs appear to be *Populus*-specific. Comparative analyses showed that genes targeted by conserved and non-conserved miRNAs are biased mainly towards development, electron transport and signal transduction processes. Similar results were found for non-conserved miRNAs from *Arabidopsis*.

**Conclusion:**

Our results suggest that while there is a conserved set of miRNAs among plant species, a large fraction of miRNAs vary among species. The non-conserved miRNAs may regulate cellular, physiological or developmental processes specific to the taxa that produce them, as appears likely to be the case for those miRNAs that have only been observed in *Populus*. Non-conserved and conserved miRNAs seem to target genes with similar biological functions indicating that similar selection pressures are acting on both types of miRNAs. The expansion in the number of most conserved miRNAs in *Populus *relative to *Arabidopsis*, may be linked to the recent genome duplication in *Populus*, the slow evolution of the *Populus *genome, or to differences in the selection pressure on duplicated miRNAs in these species.

## Background

The genus *Populus *encompasses approximately 30 species divided into 6 sections [1]. Cottonwood species are in the section *Tacamahaca*. The two North American cottonwood species *P. balsamifera *and *P. trichocarpa *are so closely related that the latter is often referred to as a subspecies, i.e. *Populus balsamifera *var.*trichocarpa *[1]. In general, gene sequences among the different *Populus *species show high similarity (>95%) [2] and as close as 99% between P. *balsamifera *and *P. trichocarpa *for the few cDNAs sequenced in P. *balsamifera*. In addition to its economic and ecological importance [3], *Populus *was chosen as a model for trees because it has a relatively small genome (500 MB), just four times the size of the *Arabidopsis *genome. Moreover, several genomic tools are available for poplars, such as detailed physical and genetic maps [3], a large number of expressed sequence tags (EST) (~116,202) [4,5]. Additionally, *Populus *grows rapidly, is easily transformed, regenerated, and propagated vegetatively [3]. The first draft of the genome sequence is now complete [3] for *Populus trichocarpa *with nearly 93% of the genome being currently assembled into chromosomes. The 7% non-assembled sequences primarily correspond to heterochromatic regions. The genome sequence for *Populus trichocarpa *facilitates functional analyses of genes in *Populus *as well as comparative and functional genomics with closely related species, especially within the Salicaceae.

MicroRNAs (miRNAs) and small interfering RNAs (siRNAs) are short (20–24 nucleotides) non-coding RNA molecules that have been demonstrated to play a key role in the regulation of gene expression [6,7]. In a pattern opposite that of siRNAs, which are generated from double-stranded RNA, miRNAs are transcribed from a long precursor molecule folded upon itself (hairpin). This precursor molecule is then cleaved by the Dicer-Like1 (DCL1) protein resulting in a miRNA:miRNA* complex, which after transport to the cytoplasm separates into the miRNA and miRNA* units [8]. One strand (miRNA) serve as a guide for the RNA-induced silencing complex (RISC), which cleave the RNA of target genes at the paired region [9]. Compared to other mechanisms that regulate gene expression, identifying a gene targeted by a miRNA is a straightforward process in plants. Since the mature miRNA and its complementary target sequence have almost perfect complementarily, identifying a miRNA usually leads to the prediction and/or identification of its target. miRNAs have been shown to target genes that are involved in development, metabolism, stress tolerance, and defense in various plant species [6,7].

A great deal of effort has gone into identification of miRNAs in the two model plants, *Arabidopsis *and rice [9-23]. Recently, two thorough analyses of *Arabidopsis *miRNAs were published [24,25]. These two studies dramatically increased the number of miRNAs identified in *Arabidopsis *from 60 to 109 families. However, miRNA identification in *Populus *has been limited compared to *Arabidopsis*. To date, there has been only one exhaustive study [26] in which 22 miRNAs expressed primarily in wood development and stress resistance were identified. Eleven of these miRNAs are conserved in other plant species and ten are absent from *Arabidopsis*. In total, 33 miRNA families represented by one or a few loci in *Populus *are reported in miRBase (Release 9.1) to date. In contrast, 109 and 62 families were reported for *Arabidopsis *and rice, respectively. Moreover, most of the newly identified *Arabidopsis *miRNA families [24,25] are not conserved in *Populus *and rice. A similar situation was found for rice and *Populus *miRNAs where 31 and 11 "species-specific" (i.e. found only in one species to date) families were identified (miRBase, release 9.1; [26], respectively. The large number of species-specific miRNAs raises questions about their function. Are these miRNAs all functional? Are they controlling the expression of species-specific genes? These questions stress the importance of completing the catalog of miRNAs in *Populus *by deep sequencing, identifying *Populus*-specific families, and analyzing their evolution. Identifying the targets of *Populus*-specific miRNAs will also help discover their functional roles in the diversification of *Populus *phenotypes and adaptation to different climates. Furthermore, comparing miRNA diversity between *Populus*, a member of the eurosid I clade, and *Arabidopsis*, a member of the eurosid II clade, will help to determine the set of miRNAs that have diverged or have been lost in these two clades since the divergence from their common ancestor [27,28] Moreover, comparing miRNA distribution and diversity in an annual plant (*Arabidopsis*) and a perennial plant (*Populus*), which have different life cycles, different developmental and physiological patterns, as well as different ecological distributions, should help to identify miRNAs that have diverged and might be involved in functions specific to annual versus perennial plants.

Until recently, most experimental miRNA isolation studies involved cloning and capillary sequencing. The concatamerization of sRNA clones, followed by cloning and cDNA isolation from bacteria before sequencing make this approach laborious and costly. Moreover, most of the miRNAs identified using this approach are highly expressed. The recently introduced 454 ultrahigh throughput sequencing technology [29] provides a better alternative. This technology generates millions of bases per run and has been used successfully for sequencing the genomes of bacteria [30], chloroplasts [31], and mitochondria [32], as well as for transcriptome analyses [33]. It was also used recently for sRNA sequencing in *Arabidopsis *and the basal eudicot *Eschscholzia californica *[21,24,25,28]. In these studies, the number of miRNAs identified in *Arabidopsis *doubled the number previously discovered in total from over 30 studies using capillary sequencing. The greater efficiency of discovery, including variants that are expressed at low levels, derives from the much deeper coverage of the sRNA population provided by pyrosequencing, and avoidance of cloning in the 454 system.

Here, we used 454 pyrosequencing [29] of small RNA libraries isolated from leaf and vegetative bud tissues in *Populus balsamifera*. *P. balsamifera *was chosen for this study because of the local availability of trees for this and future studies, the very close relationship of *Populus balsamifera *and *P. trichocarpa *[2], the large amount of genetic variation among *P. trichocarpa *trees [1,3], and the fact that the *P. trichocarpa *tree for which the genome sequence was obtained is no longer alive. We identified 123 new loci of previously reported miRNA families and 61 new non-conserved, unique miRNA sequences belonging to 48 families. We compare the distribution of these miRNA sequences in *Populus *with miRNA sequences from other land plants and discuss their evolution. Targets of these new miRNAs were predicted, including genes involved in development, resistance to biotic and abiotic stresses, and other cellular processes.

## Results

### sRNA sequence analysis

**Figure 1:**
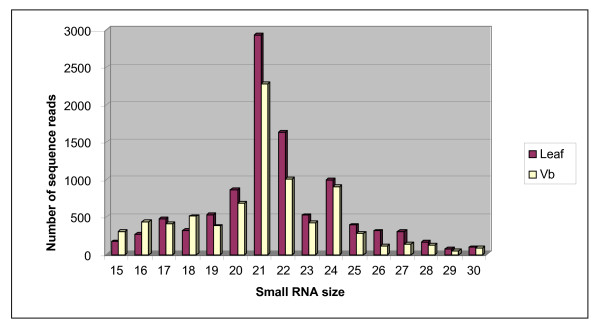
Size distribution of unique sRNA sequences obtained from leaf and vegetative bud (Vb) tissues of *Populus balsamifera *by pyrosequencing. Length of small RNAs is given on the x-axis in base pairs. Abundance of sRNA lengths were determined from the total number of high quality 454 reads after removal of redundant sequences but prior to selection for complete matches to the poplar genome sequence.

Pyrosequencing of small RNAs from *Populus *leaves and vegetative buds generated 41,323 and 35,572 reads respectively. Of these, 36,841 and 31,574 sequences from leaves and vegetative buds, respectively, were complete, containing the 9 nucleotides of both the 5' and 3' adapters. The set of leaf reads included 2,289 tRNAs, 6,146 snoRNAs, 11,594 chloroplast rRNA, and 6,867 mitochondrial rRNA sequences. Similar results were obtained for vegetative buds. After removal of these contaminants, a total of 14,768 and 12,264 sRNA sequences, with sizes between 15 and 30 nucleotides, remained for the leaf and bud samples. By removing redundant sequences from these two data sets, we identified 5,998 (Additional file 1) and 6,339 (Additional file 2) unique sRNA sequences from leaf and vegetative buds, respectively. Of these, a total of 2,607 and 2,167 unique sRNA sequences matched the *Populus *genome assembly; these were considered for further analysis. sRNAs of 21 nt in length were the most abundant class among the 15–30 nucleotide sequences (Fig. 1) suggesting that most of the small RNAs identified are processed by the *Populus *DCL1 homolog. For the two RNA samples obtained from vegetative buds and leaves, 1,619 and 1,876 unique sequences were obtained more than twice.

### A small set of miRNA families is differentially distributed in *Arabidopsis*, *Populus *and rice

Comparison with miRBase 9.1 [34] enabled the identification of 262 and 303 unique sequences corresponding to annotated poplar miRNAs in leaves and vegetative buds, respectively. By removing redundancy due to identical sequences from the two samples we identified 112 sequences belonging to 32 miRNA families (Additional file 3). A search for close members from these 32 families allowed us to identify 142 more members, which increased the number of conserved miRNAs identified to 254 (Additional file 3). Most of the *Populus *miRNAs reported previously [26], including nine miRNAs (miR473, miR475-477) reported only in *Populus*, were found in our dataset. Several miRNAs (miR171, miR408, miR475, miR476, miR477, miR479) that have been shown to be differentially expressed in phloem and xylem development and physical (tension and compression) stress [26], were found in vegetative buds and leaves grown under normal conditions. However, five *Populus*-specific miRNAs (miR474, miR478-miR481), including miR478, for which 19 members had been previously identified, were not found in our data.

Comparative analyses showed that 26 miRNA families previously annotated from either *Arabidopsis*, rice or *Physcomitrella *were found in our data. All of the 21 miRNAs conserved between *Arabidopsis *and rice [7,24] were also found. We also observed miR828 and miR858 in *Populus *which had previously been reported as *Arabidopsis*-specific [24]. These miRNAs, along with miR403, miR408, and miR473, increase to six the number of miRNAs shared by *Arabidopsis *and *Populus *but not found in rice. In contrast, ten miRNA families (miR413, miR414, miR415, miR416, miR417, miR418, miR419, miR420, miR426, miR435) were shared by *Arabidopsis *and rice but not found in *Populus*. miR1213 was discovered in *Physcomitrella *[35] but has not yet been found in *Arabidopsis *or rice.

### *Populus *conserved miRNAs are encoded by large gene families

**Figure 2:**
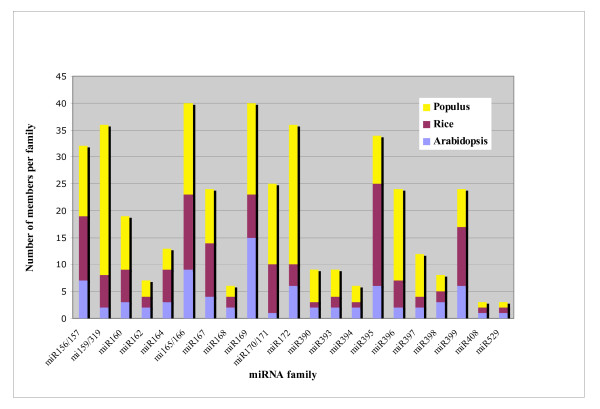
Number of paralogs (*bona fide *miRNAs as well as candidate miRNAs) identified in *Populus *in this study versus *Arabidopsis *and rice, from 21 conserved miRNA families. Note the general lack of correlation among sizes of miRNA families among the three species, with the exception of the smallest families. The *Populus *miRNAs families were determined after removal of redundant sequences and after selection for those with complete matches to the poplar genome sequence.

A query of *Populus *small RNAs against miRBAse (release 9.1) allowed us to identify 32 previously reported families (Additional file 3). Since it's common for identical mature miRNAs to be encoded in multiple paralogous loci in a single genome, we searched for all new loci corresponding to previously identified miRNAs. Indeed, we were able to map all conserved miRNA sequences on the *Populus *genome. Their flanking sequences (300 nucleotides on each side) were retrieved, aligned with *Populus *known hairpin sequences from MiRBase (Release 9.1) and the alignment checked manually. This analysis showed that, from a total of 254 loci identified by sequencing and by *in silico *analyses in this study, 131 correspond to previously reported miRNA loci (Additional file 3), while 123 are new loci. Previously un-annotated paralogs were identified for most miRNA families, with the exception of miR168 and miR408. For families miR156/157, miR159, miR319, miR162, miR172, miR396, miR397, miR473, miR475 and miR482, the number of members identified in this study was at least twice that reported previously [3,26] (Fig. 2). Analysis of the number of members per miRNA family showed that most families are expanded in size in *Populus *compared with *Arabidopsis *and rice (Fig. 2). Seven families (miR156/157, miR159, miR160, miR319, miR172, miR390, miR393, miR396 and miR397) at least doubled in size compared to the numbers previously reported for *Arabidopsis *and rice [7]. miR156/157, miR159 and miR319 are represented by 22 and 38 members respectively and three other families (miR169, miR170/171, miR165/166) are represented by more than 20 members. All of the miRNAs identified fulfilled both the phylogenetic conservation and the biogenesis criteria for miRNAs (see below) [36]. Thirty-seven new loci also fulfilled the expression criteria and can thus be considered to be *bona fide *miRNAs, while the remaining ones represent miRNA candidates for which expression remains to be confirmed.

### *Populus *non-conserved miRNAs

**Figure 3:**
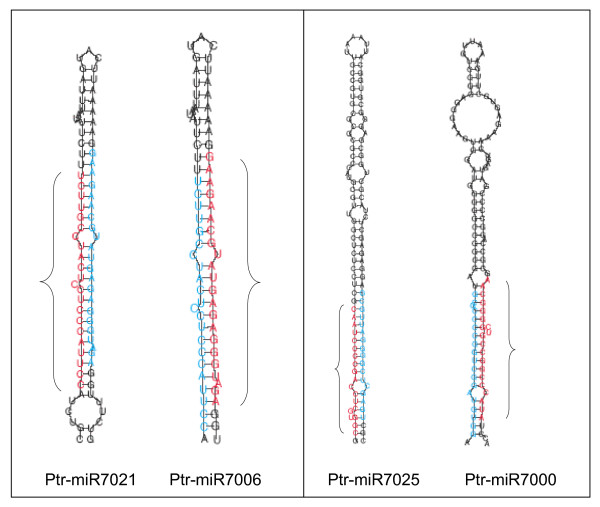
Predicted secondary structures of Ptr-miR7000, Ptr-miR7006, Ptr-miR7025 and Ptr-miR7021, newly identified non-conserved miRNAs from *Populus*. Sequences indicated in red and blue correspond to miRNAs and predicted miRNA* respectively. Brackets highlight the area offset between the miRNA sequence and the miRNA*.

**Figure 4:**
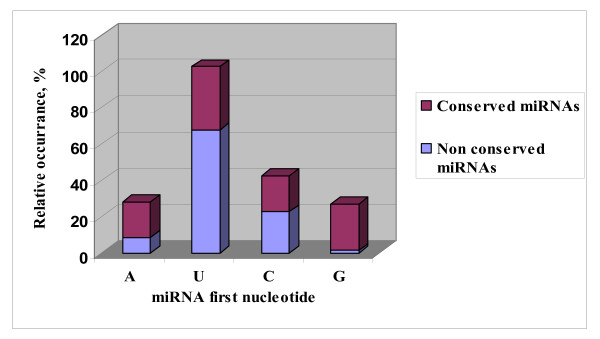
Distribution of the first nucleotide of conserved and non-conserved miRNAs determined by pyrosequencing.

Analysis of sequenced sRNA using the pipeline described in the materials and methods section identified 61 miRNA sequences unknown in *Populus *or other plant species. Analyses of the secondary structure of genes corresponding to the new miRNAs identified (see for example; Fig. 3) confirmed that they all contain features of miRNAs previously described by [16]. Forty of the miRNAs (in 34 families) were represented by more than 2 sequence reads in the sRNA data set from leaves and vegetative buds, and/or their expression has been confirmed by northern hybridization. These are thus considered as *bona fide *miRNAs by the accepted criteria of [36] (Table 1). Twenty-one sequences represented by less than two counts in the sRNA data set were considered as miRNA candidates. Comparison and distribution of the 40 miRNA sequences showed that they belong to 34 families (Table 1, Additional file 4). An exact miRNA* or a close length variant was observed for 6 of these 48 families (Table 1). The number of miRNAs identified in this study represents almost twice the number of miRNA families previously reported in *Populus *(miRBase, Release 9.1), which can now be increased to 67 families. Most of these miRNAs, including ones that are not highly expressed, start with the nucleotide "U" (Fig. 4) indicating these miRNAs have the same biogenesis origin as the conserved ones. The number of loci in each family and their chromosome locations are indicated in Table 1 (and in Additional file 4). About the same number of conserved miRNA sequences were found in vegetative buds (35 or 72%,) as in leaves (33 or 69%) (Table 1). However less than half (21 or ~44%) of the non-conserved miRNA sequences were found in both leaves and vegetative buds (Table 1).

**Table 1:** Non-conserved miRNAs and miRNA candidates identified in *Populus*.

**Families**	**Loci**	**Chrom.**	**Sequences**	**Arm**	**Len**	**VB**	**L**	miRNA*
ptr-miR7000	1	scaffold_9486	AUACCCGGCCGUCGGGGCAA	3'	20	3	0	yes
ptr-miR7001	1	LG_II	UUACCAAUACCUCUCAUGCCAA	3'	22	0	24	no
ptr-miR7002*	1	LG_VIII	UCUUUCCAACGCCUCCCAUACC	3'	22	40	73	no
ptr-miR7003	1	scaffold_163	UUCAAUGGCUCGGUCAGGUUA	3'	21	77	154	no
ptr-miR7004	1	scaffold_148	UCGUAAUGCUUCAUUCUCACAA	5'	22	39	110	no
ptr-miR7005	1	LG_VIII	UCCACAUUCGGUCAAUGUUCC	3'	21	63	50	no
ptr-miR7006*	1	LG_VIII	AGAUGGGAGAGUAUGCAAGAAG	5'	22	0	2	yes
ptr-miR7007	1	LG_XII	UUCAUUCCUCUUCCUAAAAUGG	5'	22	48	120	no
ptr-miR7008	1	scaffold_219	UCGCAAGUUGGAGGCCUGGCC	5'	21	21	0	no
ptr-miR7009-1#	5	LG_XII	UUCUGAACUCUCUCCCUCAAC	5'	21	0	2	no
ptr-miR7009-2#	5	LG_XII	UUCUGAACUCUCUCCCUCAAC	5'	21	0	2	no
ptr-miR7009-3#	5	LG_XII	UUCUGAACUCUCUCCCUCAAC	5'	21	0	2	no
ptr-miR7009-4#	5	LG_XV	UUCUGAACUCUCUCCCUCAAC	5'	21	0	2	no
ptr-miR7010#	1	LG_XV	UAAUCUCCACCAUCUCAGCUU		21	2	0	no
ptr-miR7011	1	scaffold_163	CACAAGCAAUCUAGUUGGCUC	3'	21	0	5	no
ptr-miR7012#	1	scaffold_196	AACGACUCUCGGCAACGGA	5'	19	0	2	no
Ptr-miR7013*	1	Scaffold _129	AUUCCUCUUCCUAAAAUGG	5'	19	1	1	no
ptr-miR7014#	1	LG_VIII	CUCCACAUUCGGUCAAUGUUC	3'	21	2	0	no
ptr-miR7015	1	LG_XIII	UUCCCAACUCCACCCAUCCCAU	3'	22	0	3	no
ptr-miR7016	1	scaffold_228	CCGAUUGAAUGGUCCGGUGAA	5'	21	3	5	no
ptr-miR7017	1	Scafold_131	UUUUGGUAAUGCAAGUGUUGC	3'	21	0	5	no
ptr-miR7018	1	LG_IX	UGCAUUUGCACCUGCACCUUA	5'	21	4	0	no
ptr-miR7019	1	LG_X	UGCCGACCCCACCCAUGCCAA	3'	21	37	2	no
ptr-miR7020	1	scaffold_853	GAAUGGUCCGGUGAAGUGUU	5'	20	3	0	yes
ptr-miR7021	1	LG_VIII	UCUUGCCUACUCCUCCCAUUCC	3'	22	7	10	yes
ptr-miR7022	1	scaffold_456	CGGGGUAUUGUAAGUGGCA	5'	19	3	0	yes
ptr-miR7023	1	LG_V	AAUCUCCACCAUCUCAGCUUC	3'	21	2	2	no
ptr-miR7024*	1	scaffold_1029	AUUCAGCCCCAUGUCGCUC	5'	19	2	0	no
ptr-miR7025	1	scaffold_20519	CAAUCCCCGACCUCGUGGC	3'	19	0	3	yes
ptr-miR7026#	2	LG_XIII	UCCGAUCAUUCCUCCCUCUCC	3'	21	1	1	no
ptr-miR7027#	2	Scafold_11788	UGCUGCCGAGGCCUGGCCUCC	3'	21	1	1	no
ptr-miR7028#	2	Scafold_20519	GGAGGCCAGGCCUCGGCAGCA	3'	21	1	1	no
ptr-miR7029-1*	3	LG_VII	UCUCGGACCAGGCUUCAUUCC	3'	21	32	35	no
ptr-miR7029-2*	3	LG_VIII	UCUCGGACCAGGCUUCAUUCC	3'	21	32	35	no
ptr-miR7029-3	3	LG_X	UCUCGGACCAGGCUUCAUUCC	3'	21	32	35	no
ptr-miR7030	2	LG_X	CACAUUCGGUCAACGUUCGAG	3'	21	10	8	no
ptr-miR7031-1#	4	LG_I	UGUUCAUGCUAAUUAAUUAGC	5'	21	0	2	no
ptr-miR7031-2#	4	LG_I	UGUUCAUGCUAAUUAAUUAGC	5'	21	0	2	no
ptr-miR7031-3#	4	LG_IX	UGUUCAUGCUAAUUAAUUAGC	5'	21	0	2	no
ptr-miR7031-4#	4	LG_X	UGUUCAUGCUAAUUAAUUAGC	5'	21	0	2	no
ptr-miR7032*	1	LG_X	UUGCCGACCCCACCCAUGCCAA	3'	22	37	66	no
ptr-miR7033-1*	4	LG_IV	UGGUUGUGGUUGCUUUUCAAA	3'	21	0	2	no
ptr-miR7033-2*	4	LG_IV	UGGUUGUGGUUGCUUUUCAAA	3'	21	0	2	no
ptr-miR7033-3*	4	LG_IV	UGGUUGUGGUUGCUUUUCAAA	5'	21	0	2	no
ptr-miR7033-4*	4	LG_VIII	UGGUUGUGGUUGCUUUUCAAA	3'	21	0	2	no
ptr-miR7034#	1	LG_XII	CGAGCCGAAUCAAUAUCACUC	3'	21	0	2	no
ptr-miR7035	1	LG_VIII	CUACUCCUCCCAUUCCAUCUGC	3'	22	0	4	no
ptr-miR7036	1	LG_XIV	CUCUCCCUCAAGGCUUCCAA	5'	20	3	5	no
ptr-miR7037	1	scaffold_196	UAAACGACUCUCGGCAACGGA	5'	21	4	5	no
ptr-miR7038#	1	LG_I	UGACCUUUCUUGGUGUUGUUAG	3'	22	2	0	no
ptr-miR7039	1	scaffold_163	UCAAUGGCUCGGUCAGGUUA	3'	20	3	7	no
ptr-miR7040-1#	2	LG_XIX	UUUGAUCGAUGAGGGAAUAAU	3'	21	2	0	no
ptr-miR7040-2#	2	LG_XIX	UUUGAUCGAUGAGGGAAUAAU	3'	21	2	0	no
ptr-miR7041#	1	LG_XIX	UUUGUGGAACUCGAACUGGU	5'	20	2	0	no
ptr-miR7042#	1	scaffold_163	CAGAUCAUGCCAUGACAGAAG	5'	21	2	0	no
ptr-miR7043#	1	scaffold_245	UUGGUUGCGCAUGAACCUGA	5'	20	2	0	no
ptr-miR7044	2	scaffold_163	UGACAGAAGAGUUAAAUGUUGA	5'	22	2	2	no
ptr-miR7045	2	LG_XVIII	UGCUCACUUCUCUUCUGUCAGC	3'	22	4	7	no
ptr-miR7046-1*	2	LG_VI	CCACAGCUUUCUUGAACUGCA	3'	21	2	1	no
ptr-miR7046-2*	2	LG_XVIII	CCACAGCUUUCUUGAACUGCA	3'	21	2	1	no
ptr-miR7047	1	LG_XIV	UUGACGAAAUGUGACGACUAC	3'	21	7	0	no

The length (len) of each miRNA, the number of loci (loci), the number of times a sequence was sampled in leaf (L) and vegetative buds (VB), and whether or not a miRNA star (miRNA*) was observed are indicated. (*) indicate miRNAs for which the expression was confirmed by northern hybridization. (#) indicate miRNA candidates.

**Figure 5:**
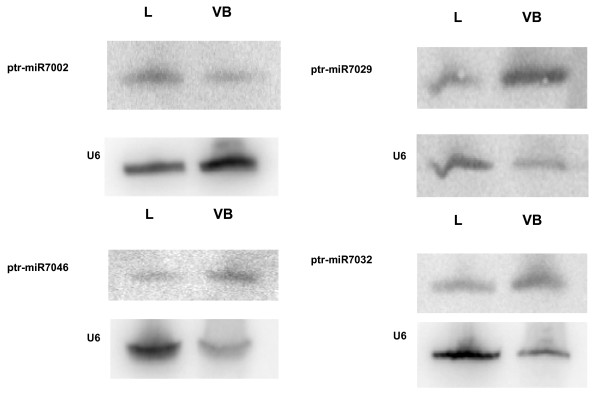
Expression analysis of four new, non-conserved miRNAs from *Populus*. Names of miRNAs are indicated on the left side. The results of by northern hybridization are show for four arbitrarily chosen genes as a validation of the sequencing results. The U6 small nuclear RNA (snRNA) was used as loading control. L and VB correspond to leaf and vegetative bud tissues respectively.

Relative expression of the non-conserved miRNAs varied widely, based on the number of sequences observed for each miRNA in our dataset. The three (~4%) most highly expressed miRNAs (7003, 7004, 7007) were represented by more than 100 sequences in leaves, and 48–77 times in vegetative buds. Nine (18%) of the non-conserved miRNA families were present between 20 and 100 times, while the remaining 37 miRNA families had lower levels of expression. Approximately 34% of non-conserved miRNA families were represented by more than 5 sequences in our dataset, of which six miRNAs (miR7002, miR7003, miR7004, miR7005, miR7007, and miR7032) were expressed relatively highly in leaves (50 or more times) while only two were observed at those levels in vegetative buds (miR7003 and miR7005). To validate the sequencing results, the expression was confirmed for 4 arbitrarily chosen genes representing 9% of these families by northern hybridization (Fig. 5).

### Target search of non-conserved miRNAs

**Figure 6:**
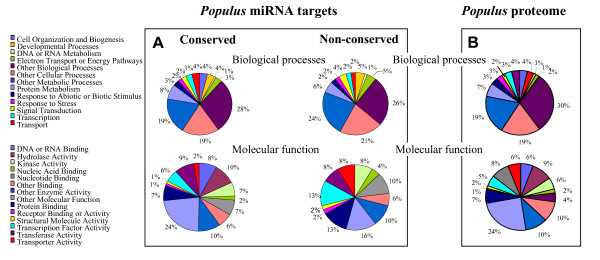
Pie chart representation of Gene Ontology classification of putative molecular functions of the *Populus *predicted genes and miRNA predicted targets as well as biological processes in which they are involved. See text for GOstat tests of significance.

In order to predict potential regulatory targets of non-conserved miRNAs, a search was performed on the TIGR *Populus *cDNA dataset as described in the methods section. For 17 (35%) of the non-conserved miRNA families, no putative target could be predicted on *Populus *CDSs and cDNAs. In total, putative targets were predicted for 31 (65%) of the non-conserved miRNA families (Table 1). We used the highest scoring *Arabidopsis *BLASTP hit to annotate the putative functional category of the predicted target genes (Fig. 6). For 16 (33%) miRNA families, the target search allowed *Populus *unigenes that have no homology to known *Arabidopsis *sequences to be identified. Twenty-six (54%) of the non-conserved miRNAs had a best alignment to *Arabidopsis *sequences, of which two predicted target genes were annotated only as expressed sequences. Eleven (23%) miRNA families have more than one predicted target, 5 of which have more than 3 predicted targets. Approximately 13% of the target genes predicted (Table 2) encode transcription factor proteins involved in various processes of plant development such as MYB, homeodomain-leucine zipper, ANAC (abscisic-acid-responsive), and No Apical Meristem (NAM). Predicted target genes that encode for transcription factors and DNA and RNA binding represented 13% of the miRNAs. Several other predicted targets include genes involved in resistance to biotic and abiotic stresses such as CC-NBS-LRR, TIR-NBS-LRR, Calmodulin-binding protein, cyclic nucleotide-gated channel C, as well as trypsin and protease inhibitor family proteins (Kunitz family). Three miRNAs predicted target genes encoding polyphenol oxidase, which belongs to the lignin synthesis pathway. Another predicted target gene encodes dihydroquinate dehydratase protein in the shikimate pathway. Targets involved in other cellular and developmental processes, such as transport, were also identified (Table 2).

**Table 2:** Putative target genes of non-conserved miRNAs and miRNA candidates identified in *Populus*.

**miRNA Family**	***Populus *Predicted Target**	**Homolog in *Arabidopsis***	**Target Predicted Function**
**miRNA with target predicted in *Populus *with homolog in *Arabidopsis***			

ptr-miR7000*	CX173939	At4g12800.1	Photosystem I reaction center subunit
ptr-miR7001	DT522234	At1g12230.1	Transaldolase ToTAL2
ptr-miR7002	TA2404_3696	At3g54190	Hypothetical protein
ptr-miR7006	TA2404_3696	At3g54190	Hypothetical protein
ptr-miR7007	Grail3.0035010701	At4g17980	No apical meristem (NAM)
	EstExt_fgenesh4_pg.C_LG_II1399	At1g33060	Transcription factor (ANAC014)
ptr-miR7009-1	Eugene3.00060403	At2g41450	PAXIP1L protein
	Fgenesh4_pg.C_LG_XII000915	At1g66350	Scarecrow transcription factor
ptr-miR7009-2	Eugene3.00060403	At2g41450	PAXIP1L protein
	Fgenesh4_pg.C_LG_XII000915	At1g66350	Scarecrow transcription factor
ptr-miR7009-3	Eugene3.00060403	At2g41450	PAXIP1L protein
	Fgenesh4_pg.C_LG_XII000915	At1g66350	Scarecrow transcription factor
ptr-miR7009-4	Eugene3.00060403	At2g41450	PAXIP1L protein
	Fgenesh4_pg.C_LG_XII000915	At1g66350	Scarecrow transcription factor
ptr-miR7010	Gw1.X.2191.1	At3g12530	DNA replication protein-related
	Gw1.41.327.1	At4g09350	DNAJ heat shock
ptr-miR7013	EstExt_Genewise1_v1.C_LG_V0549	At1g34190	No apical meristem (NAM)
	Fgenesh4_pm.C_scaffold_29000148	At2g38250	DNA-binding protein-related
	Eugene3.00110906	At1g53140	Dynamin family protein
	EstExt_fgenesh4_pg.C_LG_II1399	At1g33060	Transcription factor (ANAC014)
	EstExt_fgenesh4_pg.C_LG_XIV0205	At4g17980	Transcription factor (ANAC071)
ptr-miR7015	Fgenesh4_pg.C_LG_XVIII000967	At2g24300	Calmodulin-binding protein
	Gw1.245.24.1	At5g36930	Toll-Interleukin-Resistance (TIR)
	EstExt_Genewise1_v1.C_LG_XVIII0864	At5g11790	Ndr family protein
	Eugene3.00190231	At4g27220	ATP binding
	Gw1.245.9.1	At5g36930	TIR-NBS-LRR
	Gw1.3272.9.1	At5g36930	TIR-NBS-LRR
	Gw1.I.4710.1	At5g36930	TIR-NBS-LRR
	Gw1.XI.1412.1	At5g36930	TIR-NBS-LRR
	Gw1.XI.1580.1	At5g36930	TIR-NBS-LRR
ptr-miR7018	Grail3.0033032901	At2g45620	Nucleotidyltransferase
	Fgenesh4_pg.C_LG_VIII001448	At5g43630	Nucleic acid binding
ptr-miR7021	Gw1.117.167.1	At5g36930	TIR-NBS-LRR class
ptr-miR7022	TA14145_3694	At5g41680.2	Putative senescence-associated
ptr-miR7023	CN519776	At5g15270.1	KH domain-containing protein
	Eugene3.00041046	At5g15270	KH domain-containing protein
	TA578_3690	At4g09350	Hypothetical protein
	Fgenesh4_pg.C_scaffold_70000157	At3g06350	Dehydroquinate dehydratase
	Gw1.41.327.1	At4g09350	DNAJ heat shock
	Gw1.I.8684.1	At4g23740	Kinase protein
	Gw1.X.2191.1	At3g12530	DNA replication protein Psf2
ptr-miR7024	EstExt_fgenesh4_pg.C_LG_IX0044	At1g76310	B-like cyclin
	EstExt_fgenesh4_pg.C_LG_XVI0015	At5g47630	Acyl carrier family protein (ACP)
	Grail3.0083002901	At5g49980	F-box
ptr-miR7025	DN496261	At3g19830	C2 domain-containing protein
ptr-miR7032	TA4511_293756	At3g05560.1	60S ribosomal protein
ptr-miR7033-1	Fgenesh4_pg.C_LG_III000186	At5g57010	Calmodulin-binding family protein
ptr-miR7033-2	Fgenesh4_pg.C_LG_III000186	At5g57010	Calmodulin-binding family protein
ptr-miR7033-3	Fgenesh4_pg.C_LG_III000186	At5g57010	Calmodulin-binding family protein
ptr-miR7033-4	Fgenesh4_pg.C_LG_III000186	At5g57010	Calmodulin-binding family protein
ptr-miR7034	Fgenesh4_pg.C_LG_XII000915	At4g08250	Scarecrow transcription factor
ptr-miR7035	Eugene3.00020435	At4g30720	Expressed protein
ptr-miR7036	Eugene3.00020435	At3g63390	Expressed protein
ptr-miR7040-1*	TA11116_113636	At4g27220.1	NBS-LRR type disease resistance
ptr-miR7040-2*	TA11116_113636	At4g27220.1	NBS-LRR type disease resistance
ptr-miR7041	CK096204	At5g53130.1	Cyclic nucleotide-gated channel C
	TA14492_3694	At5g28450.1	Chlorophyll a-b binding 7
	TA16704_3694	At1g80550	Hypothetical protein
	TA20868_47664	At5g53130.1	Cyclic nucleotide-gated channel C
ptr-miR7042	CK092266	At5g27380.1	Glutathione synthetase
ptr-miR7044	TA1634_80863	At3g59480.1	Putative fructokinase 2
ptr-miR7046-1	EstExt_Genewise1_v1.C_1840054	At1g17860	Trypsin and protease inhibitor
ptr-miR7046-2	EstExt_Genewise1_v1.C_1840054	At1g17860	Trypsin and protease inhibitor

**miRNA with annotated target predicted in *Populus *with no homolog in *Arabidopsis***			

ptr-miR7005**	AJ777690	no	Polyphenol oxidase
	Eugene3.00100932	no	Polyphenol oxidase
ptr-miR7012	CK110871	no	Hypothetical protein
ptr-miR7014**	AJ777690	no	Polyphenol oxidase
ptr-miR7016	AJ773830	no	Hypothetical protein
	TA3964_3691	no	Ac1147-like protein
	TA7847_3695	no	CDH1-D
ptr-miR7017	Gnl|PPLGI|CK106645	no	Hypothetical protein
ptr-miR7019	Gnl|PPLGI|TC19799	no	Histidine kinase like ATPase
ptr-miR7020	AJ773830	no	Hypothetical protein
	TA3964_3691	no	Ac1147-like protein
	TA7847_3695	no	CDH1-D [Gallus gallus (Chicken)]
ptr-miR7030	AJ777857	no	Polyphenol oxidase
	DN483929	no	Polyphenol oxidase
	TA14535_3695	no	Polyphenol oxidase
ptr-miR7037	TA12949_3694	no	Hypothetical protein ART3

**miRNA with target predicted non-annotated in *Populus *with no homolog in *Arabidopsis***			

ptr-miR7003	Gnl|PPLGI|TC35094	no	-
ptr-miR7004	BU871048	no	-
ptr-miR7014	Eugene3.89590001	no	-
ptr-miR7026	DN487711	no	-
ptr-miR7029-1	CV259704	no	-
	CV259944	no	-
	DT491789	no	-
ptr-miR7029-2	CV259704	no	-
	CV259944	no	-
	DT491789	no	-
ptr-miR7029-3	CV259704	no	-
	CV259944	no	-
	DT491789	no	-
ptr-miR7039	CV230379	no	-
ptr-miR7047	BU873871	no	-

"-" indicates that no target was found in *Populus*. "no" indicates that no *Arabidopsis *homolog of the *Populus *target was found. Other target were found for some miRNAs, but they have no significant similarity either to *Arabidopsis *(*) or other plant (**) protein sequences.

## Discussion

### *Populus *contains at least 100 miRNA families

Previous studies [26,3] identified 33 miRNA families (miRBase, release 9.1) in *Populus*. In this study, 254 conserved miRNA loci belonging to 38 families and 40 non-conserved miRNA loci representing 34 new families were also identified. Moreover, 21 miRNA candidates belonging to 14 families were identified. This increased the number of miRNAs identified in *Populus *by almost 200%. Among the newly identified miRNAs, 123 correspond to new loci of previously identified miRNA families [26,3]. The other 61 miRNA families showed no sequence conservation with miRNAs from *Arabidopsis*, rice, or other plant sequences in miRBase. Some of these non-conserved sequences may have resulted from sequencing errors [29,31], but almost all (99%) of them were captured repeatedly. Moreover, rarely did these variants contain mononucleotide runs that are the expected source of most 454 errors [29,31]. Furthermore, because we analyzed only those sequences that exactly matched the *Populus *genome, most reads with sequencing errors would have been removed and not considered as miRNAs in this study. The sequences generated, both conserved and non-conserved, increased the total number of miRNA families in *Populus *by 67 families. Because many members of these 67 miRNA families were identified by genome-scale data mining and sequencing, it is possible that most of the miRNAs in *Populus *have now been discovered. However, because we used 100% homology to the *P. trichocarpa *genome sequence to identify miRNAs, there may be additional miRNAs in *P. balsamifera *that we missed due to slight sequence divergence from *P. trichocarpa*. Indeed, the sequences of the very few *P. balsamifera *ESTs in GenBank differ from *P. trichocarpa *in the 1 – 2% range (data not shown), including possible sequencing errors. Also, the fact that that some miRNAs were observed only twice indicates that even deeper sequencing might still capture new miRNAs. Deeper sequencing is also likely to isolate more miRNA* sequences for the newly identified miRNAs, as miRNA* was identified for only six of the non-conserved miRNAs. The low number of miRNA* identified is probably due to non-saturating coverage of the small RNA libraries. Also, sampling of other tissues at different developmental stages, or in response to physiological conditions, may result in the identification of new non-conserved miRNA families, their corresponding members, and miRNA* sequences.

In summary, the composition of the miRNA pool in *Populus *seems to be similar to that in *Arabidopsis *and rice, though they differ substantially in family number and in the occurrence of many lineage specific miRNAs. Indeed, *Populus*, *Arabidopsis*, and rice contain 21 conserved families as well as large sets of non-conserved miRNAs. The identification of such a large number of miRNAs from *Populus *represents a key resource for comparative and functional analyses of miRNAs as well as the study of their evolution.

### Differential conservation of miRNAs between *Populus*, *Arabidopsis *and rice

All 21 families conserved between *Arabidopsis*, *Populus *and rice [7] were identified in our dataset. The conservation of this set of miRNA families in such taxonomically divergent land plant species (*Arabidopsis*, *Populus*, rice and *Physcomitrella*) indicates these miRNAs are subject to functional constraints which keep them highly conserved across species. However, a small set of miRNAs seems to be differentially distributed in *Arabidopsis*, *Populus *and rice. We found 2 *Arabidopsis *(miR828 and miR858) and one *Physcomitrella *(miR1213) miRNA families that were not previously reported in *Populus*. This extends the number of conserved miRNAs between *Arabidopsis *and *Populus *to 24 families. Furthermore, 10 *Arabidopsis *miRNA families (miR413-435) which have been reported as conserved between *Arabidopsis *and rice were not found in our sequence data. These sequences can not be identified in the *Populus *genome and cDNA databases either, even under relaxed alignment parameters. This indicates that the conservation of some miRNAs between species is not only associated with the phylogenetic distance between the species but may also be under the control of other undetermined constraint(s).

### Organization and evolution of *Populus *miRNAs

Comparison of the numbers of paralogous miRNA loci per family in *Arabidopsis*, *Populus *and rice indicate that most conserved miRNA families have different numbers of paralogs in the different lineages. The number of loci per miRNA family is generally higher in *Populus *compared to *Arabidopsis *and rice. Several *Arabidopsis *and rice families such as miR156/157, miR159/319, miR162, miR172, miR396, miR397, miR473, and miR475 are nearly double in size in *Populus*. This result is not in accord with an initial report [7] that the miRNA gene family size is similar between the three model species (*Arabidopsis*, rice, and *Populus*). More miRNA gene duplicates appear to be retained in *Populus *in parallel with the greater number of genes that have been maintained since the last genome duplication (1.4 to 1.6 genes in *Populus *per gene in *Arabidopsis*; [3]). This increase in miRNA family size is probably not be due to a difference in the level of genome duplication alone, however, since both *Arabidopsis *and *Populus *have extensive genome sequence duplication [37] and the *Arabidopsis *and rice genomes appear to have changed much faster relative to *Populus *in the time since their last common ancestor [3]. An alternative hypothesis is that there is a difference in the selection pressure on duplicated miRNAs in *Populus *and that having more miRNA members might be advantageous for adaptation to perennial growth and to different ecological environments.

### Most of the newly identified *Populus *miRNA families are not conserved

Most (92%) of the *Arabidopsis *miRNAs and miRNA candidates reported in recent studies [24,25] were not found in our *Populus *data. Thirty of the rice miRNA families are not found in either *Arabidopsis *or *Populus *(miRBase, release 9.1). Similarly, most of our non-conserved newly identified miRNA families appear to be specific to *Populus *at this point. The miRNAs found only in *Populus *to date could be relatively young, generated by recent duplication events specific to *Populus *[25]. The identification of a large set of miRNAs specific to each species supports the hypothesis that most lineage-specific miRNAs are generated by recent duplication events or other processes specific to species or clades [24]. An alternative possibility is that they may actually be ancient miRNAs that have been under positive selection and divergence in *Populus *but have lost function in *Arabidopsis*. Depending on the age of the duplication and how strong the selection pressure is, the number of functional miRNAs may vary between species. In this study, at least one target was predicted for most of the non-conserved miRNAs identified, indicating that they may be functional. However, it has been shown in a previous report [25] that target genes could not be validated for most of the apparently Arabidopsis-specific miRNAs, even though they have a high degree of similarity with known genes. Therefore, only an experimental validation of predicted targets will allow newly identified miRNAs to be confirmed as functional, and to determine if the hypothesis proposed by [25] is generally valid. Furthermore, miRNA studies in additional non-model plant species are required to determine the extent to which non-conserved miRNAs identified to date are truly species- specific or appear in other lineages.

### The non-conserved miRNAs potentially regulate a wide variety of functions including many genes that are specific to *Populus*

An important question raised by the large number of non-conserved miRNAs found in *Populus *is the function of genes targeted by these miRNAs. Prediction of gene targets using annotated *Populus *cDNAs showed that nearly 65% of the newly identified non-conserved miRNAs have targets. The remaining 35% could correspond to genes that are not identified using automatic annotation or lowly expressed genes that are not yet represented in *Populus *cDNA and EST datasets. Alternately, some of these miRNAs may have different modes of target recognition [16]. As shown in Table 1 and Table 2, 30% of the genes targeted by non-conserved miRNAs have no best hit in *Arabidopsis *indicating that these genes could be *Populus *specific, or at least diverged in Arabidopsis. This important discovery suggests that non-conserved miRNAs are involved in the regulation of cellular, physiological or developmental processes in a manner that is specific to these species. Nine (14%) of annotated genes targeted by non-conserved miRNAs are transcription factors or nucleic acid binding proteins, all of which have been previously reported in *Arabidopsis *[6,7]. These transcription factors include *Ap2*, *MYB*, *Squamosa*, *Homeobox-leucine zipper*, and *No Apical Meristem (NAM)*. Another class of highly represented non-conserved miRNAs predicted targets in *Populus *are genes involved in plant resistance to biotic and abiotic stresses. The most common are CC-NBS-LRR genes and TIR-NBS-LRR resistance genes. This class represents the most frequently cloned resistance genes to date, which play an important role in the detection of various pathogens [38]. Other resistance gene predicted targets included hypersensitive-induced response protein (Band 7 protein family) involved in plant defense against biotrophic pathogens [39], trypsin and protease inhibitor family protein (Kunitz family,) as well as polyphenol oxidases that are involved in plant defense against herbivores and other stresses [40]. Moreover, several housekeeping genes are predicted to be targeted by the new non-conserved miRNAs.

**Figure 7:**
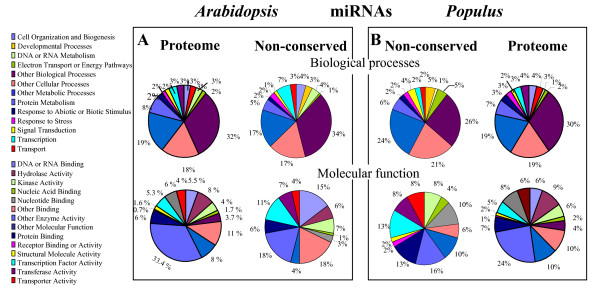
Pie chart representation of Gene Ontology classification of putative molecular functions of non-conserved miRNAs from *Arabidopsis *and *Populus *and the biological processes in which they are involved. See text for GOstat tests of significance.

To check if conserved and non-conserved miRNAs from *Populus *target genes with similar function, we compared the biological functions of their potential target genes (Fig. 6). These analyses showed that conserved and non-conserved miRNAs from *Populus *target genes are involved in the same biological processes. However, non-conserved miRNAs have twice as many putative target genes encoding transcription factors, transporters, protein binding genes, and nucleic acids binding genes as conserved miRNAs. A statistical analysis using the GOstat program [41] confirmed the bias of non-conserved miRNAs towards genes involved in two biological processes (transduction signal and transport, p-value < 0.05). Moreover, since a third of the non-conserved miRNA predicted targets had no homologs in *Arabidopsis*, no definitive conclusions could be drawn about the divergence of target genes between these two classes of miRNAs. To test if conserved and non-conserved miRNAs from *Populus *and *Arabidopsis *target genes similarly, we compared the biological functions of their predicted target genes (Fig. 7). If this hypothesis is true, miRNA targets should reflect the diversity of the *Populus *transcriptome. Our analyses showed that miRNA putative targets from *Arabidopsis *and *Populus *present similar patterns in that they were both biased toward development, transcription, DNA and RNA metabolism, protein metabolism, electron transport and signal transduction. The over-representation of these biological processes was confirmed with the GOstat program [41] (P-value < 0.02). This indicates that conserved and non-conserved miRNAs from both species are subject to similar selection pressures allowing miRNAs that regulate some biological processes to be retained.

## Conclusion

In conclusion, pyrosequencing of uncloned sRNA permitted us to make the following discoveries: (i) most members of previously reported miRNA families are also found in *Populus *leaves and buds; (ii) forty eight new miRNAs were identified that may be *Populus *specific; (iii) miRNA families are larger in size in *Populus *than in *Arabidopsis *and rice; (iv) about a third of the genes targeted by non-conserved miRNAs appear to be *Populus*-specific; (v) as in rice and *Arabidopsis*, *Populus *miRNAs primarily target genes that regulate development and that are involved in stress responses; (vi) non-conserved and conserved miRNAs target genes involved in similar biological processes; and (vii) the targets of conserved and non-conserved miRNAs are biased towards development, transcription, DNA and RNA binding, electron transport and signal transduction.

## Methods

### Tissue collection and RNA preparation

Leaves and vegetative buds were collected weekly during the months of June and July from mature *P. balsamifera *trees growing on the Pennsylvania State University campus, University Park, Pennsylvania. Total RNA was prepared by the method of Chang and collaborators [42] with modifications. Three to five grams of frozen tissue were weighed, ground to a fine powder under liquid nitrogen, and dispersed in CTAB buffer. Following 2 chloroform extractions, RNA was precipitated with LiCl_2_, again extracted with chloroform and precipitated with ethanol. The resulting RNA pellet was resuspended in 40–100 μl of DEPC-treated water, and the quality was assessed with an Agilent Technologies 2100 Bioanalyzer (Agilent Technologies). Low molecular weight or small RNAs (sRNA) were purified from total RNA by fractionation on a polyacrylamide gel.

### sRNA sequencing

Libraries of sRNA for 454 sequencing were constructed from leaf and vegetative bud RNA, without cloning, as described previously [9,28,43]. In summary, purified sRNAs were ligated to 5' and 3' adapters, reverse transcribed and amplified using Polymerase chain reaction (PCR) to produce cDNA corresponding to sRNA. The resulting cDNA was used to construct a "library" for sequencing by the addition of adaptors as per the supplier's instructions (Roche Diagnostics) and sequencing was conducted at Penn State University on an FLX model 454 DNA sequencer (454 Life Sciences) as previously described [32]. One quarter plate of 454 sequence data was obtained from each library.

### sRNA analysis and identification of *Populus balsamifera *miRNAs and their targets

The sequences produced by the FLX sequencer which corresponded to sRNA-derived cDNAs were filtered for those with 9 nucleotides of perfect match to adapter sequences at both ends, which were selected for further analyses. In addition, sRNA sequences that were not 15 to 30 nucleotides in length were discarded. sRNA sequences that passed the adapter check and size filter were then screened against chloroplast and mitochondrial genomes [44], tRNA [45], rRNA [46], snoRNA [47] and repeat sequence databases, and all contaminating rRNA, tRNA and snoRNA sequences removed. The screen for contaminant RNAs was done using BlastN with default parameters (Cutoff e-value was e-10). The cleaned sequences were then sorted by sequence identity and the relative count of each miRNA was determined. Unique sRNA sequences were queried against known miRNAs (miRBase, Release 9.1.1) using the program Patscan [48] with default parameters and two mismatches allowed to identify homologs of known miRNAs. All sequences identified were then searched against the *Populus *genome [49] to identify *Populus *homologs. Sequences for which no hit was found on the *Populus *genome, with 0 mismatches, were removed from further analysis. We then retrieved 300 nt of *Populus *genomic sequence upstream and downstream of each passed sequence and checked for secondary structures using the program MirCheck as described by [24]. Sequences that passed MirCheck were then inspected manually and were blasted against hairpin sequences from miRBase (Release 9.1.1.1) to determine which loci had been previously reported and which were new loci. A sequence was annotated as a microRNA if it fulfills the biogenesis (folding), the phylogenic conservation and/or the expression (detection by 454 sequencing) annotation criteria of Ambrose [50].

Sequences with no similarity to known miRNA sequences were used to search for non-conserved miRNAs in the *Populus *genome using Patscan with a setting of 0 mismatches, 0 insertions, and 0 deletions. Three hundred base pairs flanking the genomic positions were then retrieved and the sequences folded using RNAfold [51] and the secondary structure was checked for miRNA features using MirCheck. Sequences that passed MirCheck were sorted by their position on the chromosome and redundant sequences linked to errors of genome assembly were removed manually. Non redundant sequences having two substitutions or less were grouped in the same family. When several length variants of the same miRNA were sequenced, only variants with the highest representation were considered. The six non-conserved miRNA families for which members have identical mature miRNAs but for which expression could not be confirmed using northern hybridization, were also annotated as miRNAs. miRNA sequences were checked for conservation in the *Arabidopsis *[52] and rice (*O. sativa *ssp. *japonica *cv Niponbare; [53]) genomes using Patscan, tolerating no more than three substitutions. When a probable homolog was identified in any of these three species, the genomic sequence surrounding it was analyzed using RNAfold and the secondary structures were checked using MirCheck [16].

A search for miRNA target genes was then performed using an approach previously described [54]. All newly identified miRNA sequences were used to query the *Populus *CDS [3] and cDNA dataset [55] for potential target sequences using Patscan with default parameters and three mismatches, no insertions, and no deletions permitted. Only hits with less than two mismatches in positions 1–9, no mismatches in positions 10 and 11, and less than three mismatches after position 11 [56] in the mature miRNAs were considered good target sequences. Target sequences were then annotated using the *Arabidopsis *proteome (Blastx, e-value < 0.05). For miRNAs for which no target was identified in *Populus*, the same target search was performed on the *Arabidopsis *cDNA dataset downloaded from TIGR [56] using the same criteria to identify target sequences. Molecular functions as well as biological processes were compared for genes targeted by both conserved and non-conserved miRNAs to detect any bias in biological function. They were also compared to the whole *Populus *transcriptome. A statistical analysis, showing the probability of target enrichments in some biological processes or molecular function was conducted using the GOstat program [41] set to the following parameters: GO-DB: tair; Min Sub-GO length: 3; P-Value Cutoff: 0.1; GO-Cluster Cutoff: -1; with no correction for multiple testing because the high dependence between GO terms will cause the test to be overly conservative [41].

### miRNA expression analysis using Northern Blot

Total RNA was prepared from leaves using TRIzol reagent (Invitrogen) according to the manufacturer's recommendations with modifications. Total RNA from vegetative buds was prepared using the protocol described previously [42]. Northern blot and hybridization were done as described in [28]. In summary, total RNA was fractionated on a denaturing (urea 8%) polyacrylamide gel and transferred to a nylon membrane using a vacuum transfer system (Biorad). Probes used for hybridizations were end labeled with gamma ^32^PATP using T4 polynucleotide kinase (New England Biolabs) according to manufacturer recommendations. Non-incorporated nucleotides were removed using Centrispin-20 columns (Princeton Separations). Hybridizations were performed at 20°C below the probe melting temperature (Tm) in ULTRAhyb-Oligo (Ambion) buffer as suggested by the manufacturer. Filters were washed twice for 30 min at 20–22°C below Tm using 0.5X SSPE/0.5% SDS, exposed and scanned using a phosphoimager (Applied Biosystems).

## List of abbreviations used

ABI, Applied Biosystems; cDNA, DNA complementary to RNA; miRNA, microRNA; EST, expressed sequence tag; nt, nucleotide; PCR, polymerase chain reaction; siRNA, small interfering RNA; sRNA, small RNA; RNAi, RNA interference, Ta-siRNA, *trans*-acting-siRNA.

## Authors' contributions

AB planned the project; designed and executed the experiments, curated and analyzed the data, and supervised the work of assistants. KPW contributed extensively to the bioinformatics analyses. SDL collected *Populus *samples, participated in curating the data, and helped on northern hybridization experiments. This work was conducted in the laboratories of JC and CdP, who supported this work, contributed to the discussion of the results and also assisted in preparation of the manuscript. AB and JC wrote the paper.

## Supplementary Material

Additional File 1 Small RNA sequences identified from leaf. The length of each sequence and its occurrence were indicated. Click here for file

Additional File 2 Small RNA sequences identified from vegetative buds. The length of each sequence and its occurrence were indicated. Click here for file

Additional File 3 Conserved miRNAs identified in *Populus*. The length (len) of each miRNA, the number of times a sequence was sampled from leaf (L) and vegetative buds (VB), the chromosome location (Chr), the start (start) and the end (stop) position on the chromosome of each miRNA sequence, the miRNA orientation (Dir), and whether or not a miRNA was observed are indicated. Click here for file

Additional File 4 Non-conserved miRNAs and miRNA candidates identified in *Populus*. The sequence of each miRNA, the chromosme location of loci (chrom), the coordinate of the 300 nt flanking miRNA sequences are indicated. Click here for file
